# A Research on the Crisis Spillover Effect of Food Safety Incidents on Competitive Firms: The Influence of Political Connections and Charitable Donations

**DOI:** 10.3389/fpubh.2021.766385

**Published:** 2021-11-25

**Authors:** Rong Xiang, Mengqi Wang, Li Lin, Dongxia Wu

**Affiliations:** School of Business Administration, Zhejiang Gongshang University, Hangzhou, China

**Keywords:** crisis spillover, political connection, charitable donation, abnormal returns, food safety incidents

## Abstract

Taking the perspective of corporate social responsibility and institutional theory, this research establishes an innovative relationship between variables such as charitable donation, political connection and crisis spillover effect of firms through quantitative analysis using the event study method, regression analysis and the Heckman two-stage model. Taking 8 food safety incidents from 2011 to 2016 as research samples, this paper studies the impact of food safety incidents on the market value of both firms under crisis and their competitive firms, as well as the influence of political connection and charitable donation. Based on the current situation that the product crisis or reputation crisis of a firm will, inevitably, affect the market performance and value of its competitive firms in the same industry, this paper attempts to answer questions such as “what kind of firms are capable of minimizing this negative influence?” “will the political connection of competitive firms exert a positive or negative impact?” and “can actions taken before the crisis, such as charitable donation of competitive firms, help these firms in reducing the harm?” The conclusions are as follows: first, the occurrence of food safety incidents not only has a negative impact on the market value of the crisis firm, but also has a negative spillover effect on the competitive firm; second, charitable donations made by the competitive firm before the crisis demonstrates a positive competitive effect on the competitive firm, and the intensity of such charitable donations is positively correlated with this positive competitive effect; third, the political connection of the competitive firm has no significant impact on the crisis spillover effect. These findings provide enlightenment for the operation and management of firms in the food industry.

## Introduction

Taking the perspective of corporate social responsibility and institutional theory, this research establishes an innovative relationship between variables such as charitable donation, political connection and crisis spillover effect of firms through quantitative analysis using the event study method, regression analysis and the Heckman two-stage model. Taking 8 food safety incidents from 2011 to 2016 as research samples, this paper studies the impact of food safety incidents on the market value of both firms under crisis and their competitive firms, as well as the influence of political connection and charitable donation. The conclusions are as follows: first, the occurrence of food safety incidents not only has a negative impact on the market value of the crisis firm, but also has a negative spillover effect on the competitive firm; second, charitable donations made by the competitive firm before the crisis demonstrates a positive competitive effect on the competitive firm, and the intensity of such charitable donations is positively correlated with this positive competitive effect; third, the political connection of the competitive firm has no significant impact on the crisis spillover effect. These findings provide enlightenment for the operation and management of firms in the food industry.

As a matter of fact, crisis management of firms is a research subject that is constantly updated and iterated. Researches on individual firms are gradually extended to researches on the industry, and crisis spillover has gradually become a new research field in crisis management of firms. Faced with highly-developed social media networks and closely-connected supply chains, firms can hardly fight any battle alone in the context of industrial crisis. This paper mainly explores the following four major issues as regard to this circumstance. First, how would market value of the firms under crisis change with the occurrence of food safety incidents. Second, with such incidents happening, whether competitive firms will be affected by the crisis spillover effect; and if so, will contagion effect or competition effect take place? Third, how would competitive firms' political connection influence such crisis spillover effect? And finally, how would charitable donations made by competitive firms impact the crisis spillover effect?

## Hypotheses

### Food Safety Incidents and Market Value of Firms

It is found that the biggest and most harmful food safety incident in American history happened in the well-known fast-food chain Jack-in-the-box and has caused illnesses to more than 700 people and death of 4 children across America, due to excessive addition of Escherichia coli in food. This fast-food chain has not only lost tens of billions of dollars in sales, but also continued to pay the price for such incidents many years later (1). The event study method is often used to study stock market changes during the crisis window, which is measured by the abnormal rate of return (hereinafter referred to as AR). During the crisis, AR is the difference between the expected rate of return and the actual rate of return, which reflects the positive or negative direction of the spillover effect (2–4). A positive AR shows that the event is satisfactory and the future rate of return of the firm is also positive. While a negative AR means that the event is not welcomed and the firm's earnings will be negatively affected in the future. Extant studies have shown that, food safety incidents, in general, will lead to negative impact on the market value of the crisis firm and will also cause positive or negative influence to the competitive firms depending on different scenarios (5, 6). Generally speaking, crisis spillover effect can be further divided into the competition effect, which is positive, and the contagion effect, which is negative. Contagion effect refers to the damage to the market value of peer firms due to the impact of crisis events. On the contrary, competition effect refers to the profits gained by firms in the same industry due to the crisis of the competitors (7). According to the categorization and priming theories, a high degree of overlap fosters assimilation and a low degree of overlap fosters contrast (8). So, when a crisis occurs, the similarity between the firms involved in the incident and other firms will affect consumers' activation of either the assimilation effect or contrast effect in their brains. If the similarity between the two brands is small, the contrast effect will be activated, which will trigger a positive spillover effect; on the contrary, if the similary range of the two is large, the assimilation effect is more likely to be activated, which will cause negative spillover effect, that is, the contagion effect. And it is usually acknowledged that the similarity among competitive firms in the food industry is fairly high. Therefore, the following hypotheses are proposed.

H1a: Incident of food safety crisis has a negative impact on the market value of the crisis firm.
H1b: With the passage of the incident, its negative impact on the market value of the crisis firm generally shows a weakening trend.
H1c: The occurrence of food safety incident creates crisis spillover effect on the competitive firms, and the contagion effect is greater than the competition effect.

### The Relationship Between Political Connection and Crisis Spillover

First, the relationship between political connection and crisis spillover is two-sided. On one hand, political connection may relieve the contagion effect of the crisis spillover. When a crisis occurs to the firm, investors may tend to believe that firms with political connection will be protected by the government from being involved in the crisis (9). Similarly, investors may believe that political ties may help competitive firms gain competitiveness in the presence of crisis spillover (10). On the other hand, political ties may aggravate the contagion effect of the crisis spillover. A large number of studies have shown that firms can benefit from political ties in that it is easier for firms with political ties to obtain scarce resources and legitimacy. Also, firms with political ties will be paid more attention by investors in the market. And once concerned by more people in the market, these firms will be criticized more, which makes it easier to form a contagion effect and affect the performance of these firms.

Second, firms will usually adopt strategies that are in consistency with their own institutional advantages. There is a mutually dependent relationship between politically connected firms and the government. In order to achieve its economic goals, the government will exert pressure on the firms, and in the meanwhile, it will also deregulate or offer certain preferential policies to such firms. In this scenario, consumers' perception of the product quality of the firm will be affected by the deviation of its political connection, which will create a bad impression of the firm. In addition, people tend to have a natural distrust of politically connected firms and believe that such firms are likely to engage in unethical behaviors (9). The crisis spillover effect mentioned above is directly reflected in the average cumulative abnormal rate of return of the competitive firms. Therefore, the following hypotheses are proposed.

H2a: Political connection of the competitive firm has no significant impact on the average cumulative abnormal rate of return of the competitive firm.
H2b: Political connection of the competitive firm has a positive impact on the average cumulative abnormal rate of return of the competitive firm.
H2c: Political connection of the competitive firm has a negative impact on the average cumulative abnormal rate of return of the competitive firm.

### The Relationship Between Charitable Donation and Crisis Spillover

Charitable donation is an important part of corporate social responsibility, while the demonstration of corporate social responsibility is very likely to weaken the negative spillover effect of the crisis (11). On one hand, actions related to corporate social responsibility taken before the crisis shows that the firm has some idle resources. Generally speaking, the more charitable donations a firm makes, the more idle resources it would have (12). After the occurrence of the crisis, for the unaffected competitive firm, its stock price will decrease if it is found to be related to such negative crisis. Therefore, before the occurrence of the crisis, the idle resource signal sent by the competitive firm demonstrated through its corporate charitable donation is not enough to weaken the spillover effect of the negative crisis incident. On the other hand, corporate social responsibility has a strong relationship with consumer attribution, which will affect consumers' purchase and evaluation of the products of the affected firm (13). No matter how consumers attribute the crisis, the action of charitable donation of the competitive firm before the crisis plays an important role in reducing the crisis spillover effect (2).

A crisis may be attributed to factors related to moral issues or capability issues. A moral-issue related crisis involves acts of firms that are contrary to the existing moral standards of consumers, such as those with dishonest conducts; while a capability-related crisis involves products offered by the firm which cannot meet the perceived expectations of consumers. If the crisis incident is considered to be a moral crisis, the halo effect formed by the previous actions of the competitive firm representing its corporate social responsibilities will be helpful in separating it from the impact of the crisis. If the crisis is attributed to capability issues, then, charitable donations can effectively alleviate the crisis spillover effect by affecting consumers' perception of the brand or firm under crisis. Some scholars have proposed that consumers' evaluation of the brand or firm under crisis will form a hypothesis-confirming context, and it is based on this background knowledge that consumers will understand the crisis; thus, this hypothesis-confirming context will help reduce the negative evaluation of the brand or the firm (14). More importantly, it is worth mentioning that these evaluations generally are made based on actions related to corporate social responsibilities (15).

Charitable donation is an important act representing corporate social responsibility, which affects consumers' attribution, and in turn, consumers' perception of the brand and the firm. Once a crisis occurs, due to information asymmetry in the market, consumers tend to use their existing rigid knowledge to evaluate relevant competitive firms, which will indirectly affect the spillover effect of the crisis (16). The crisis spillover effect described above is directly reflected by the average cumulative abnormal rate of return of the competitive firm. Therefore, the following hypotheses are put forward.

H3a: Charitable donation made by the competitive firm before the crisis will positively influence the average cumulative abnormal rate of return of the competitive firm.
H3b: The intensity of the charitable donation made by the competitive firm before the crisis has a significant influence on the average cumulative abnormal rate of return of the competitive firm.
H3c: The greater the intensity of the charitable donation made by the competitive firm before the crisis, the greater the positive influence it will have on the average cumulative abnormal rate of return of the competitive firm.

## Research Design

### Sample Selection and Data Source

This paper takes 8 firms under crisis related to food safety incidents as the research object, while selecting their corresponding competitive firms, respectively, with the same or similar range of business operations, and studies the spillover effect of the food safety incidents based on the stock price changes of both the crisis firms and the competitive firms.

After sorting out news reports and government regulatory releases on food safety incidents in the food industry from 2010 to 2016, and excluding food safety incidents of non-listed companies and non-mainland A-share listed companies, eight food safety incidents of listed companies were eventually selected as the research object, with a total of 70 research samples.

### Variables

Three kinds of variables are involved in this study, namely, the core independent variables, dependent variables and control variables. Based on the extant literature, these three types of variables, which are used in subsequent empirical study, are defined as below. Table 1 provides the description of each specific variable.

**Table 1:** Description of the research variables.

**Type**	**Name**	**Measurement**
Core independent variables	If the firm has donated (ifDon)	Take the amount of charitable donations of the firm in the previous year. If there are donations, assign 1; if there is no donation, assign 0.
	Intensity of charitable donation (Don)	Take the natural logarithm of the donation expenditure out of the non-operating expenditure from the annual report data of the listed company in the previous year
	Political connection (Pol)	Take whether the key executives (chairman or general manager) of the company currently or previously served in the central government, local government, military, CPPCC (Chinese People's Political Consultative Conference) and National People's Congress in the year of the incident. If yes, assign 1; if no, assign 0.
Dependent variables	Average cumulative abnormal rate of return of the competitive firm (PCAR)	AR = ER-Actual rate of return
		CAR = ΣAR
		CAR = CAR/N
Control variables	Firm size (size)	The natural logarithm of total assets at the end of the previous year
	Asset liability ratio (debt)	Percentage of total corporate liabilities in total corporate assets in the annual report of the previous year
	Cashflow (CF)	Cash and cash equivalents/total operating income in the previous year's annual report
	Ownership concentration (OC)	Shareholding ratio of top ten shareholders in the previous year
	Incident identifier (incident)	Assign values of 1, 2, 3, 4, 5, 6, 7, and 8 to each specific incident

## Empirical Test and Result Analysis

### Quantitative Analysis Based on the Event Study Method

#### Defining the Events

According to the extant literature, the event study method typically applies to two main types of events. The first type of events in all samples are of the same nature, but maybe with different dates of occurrence. The second type is a single event, that is, the events and times to be studied in all samples are the same. This paper studies eight food safety events in crisis firms, all of which are associated with the spillover effects of food safety events. So they are typically similar events, but with inconsistent time of occurrence.

#### Defining the Estimation Period and Window Period

The estimation period and time window period need to be defined to reflect the influence of events with the changes of stock prices during the studied period. The date of occurrence of the event should be included in the event period, which includes a period of time before and after the event. The stock price some time before the event can capture the responses of the capital market on the eve of the event, which may affect the stock price due to leakage of information and other reasons. The stock price for a period of time after the event can clearly reflect the attitude of the capital market toward such event. The estimation period is defined to estimate the rate of return before the event occurs by using the data of the estimation period. The expected rate of return is subtracted from the actual rate of return after the event to obtain the abnormal rate of return brought by the event. In this study, the event window period is defined as 21 days before and after the event day, i.e., the event window is [- 10,10], and the estimation period is defined as 70 days to 11 days before the event, i.e., the estimation window is [−70, −11].

#### Determining the Research Object

This paper takes 8 A-share listed firms encountering crisis with food safety events as the main research object, and studies the spillover effect of food safety events based on the stock price changes of crisis firms and competitive firms by associating the crisis firms with different numbers of competitive firms within the same or similar business. After excluding issues related to incomplete data and so on, 70 research samples were eventually obtained according to classification based on the similarity of main businesses among the firms.

#### Calculation of Expected Rate of Return

Event study method is a research method used to analyze the impact of specific events on the company's stock price, which offers a means to measure the change of the firm value. In a rational financial market, the impact of an event will be immediately reflected in the stock price, so the impact of the event can often be considered through the change of the stock's return on equity (17). Usually, an appropriate estimation model needs to be selected and the stock price information in the estimation period needs to be used to determine the expected rate of return. According to the extant literature, there are mainly three estimation models, namely, the mean-adjusted returns model, the marketing-adjusted returns model and the OLS market model. This study adopts the market model to carry out the subsequent research.

The market model is the most complex one among the three models, and it is also the most widely used research model in the event study method (18, 19). The market model assumes that there is a stable linear relationship between market return and individual stock return. The market model is based on the data in the estimation period [−70, −11] and the event window period [−10, 10], and the regression model is established using the least square method (see as follows).


$$\begin{array}{lll} R_{it} & = & \alpha_{i}+\beta_{i}R_{mt}+\xi_{it}\\ E\left(\xi_{it}\right) & = & 0\;Var\;\left(\xi_{it}\right)=\sigma_{\xi_{it}}^{2} \end{array}$$

In the equations shown above, R_*it*_ is the expected return of stock i on day t; R_mt_ is the market rate of return on day t;α_*i*_ and β_*i*_ are both estimating parameters; ξ_it_ is the residual term. In this paper, different indicators were selected to represent the market rate of return according to different indicators actually used by different exchanges for the listed companies. To be specific, for listed companies in Shanghai Stock Exchange, Shanghai composite index rate of return is used as the indicator of the market rate of return; and for listed companies in Shenzhen Stock Exchange, Shenzhen component index rate of return is used as the indicator of the market rate of return.

#### Calculation of Abnormal Rate of Return

In the event study method, calculating the abnormal rate of return of stocks is a key step. Abnormal rate of return refers to the difference between the actual rate of return and the expected rate of return, which is shown as follows.


$$\begin{array}{l} AR_{it}=R_{it}-E\left(R_{it}\right) \end{array}$$

In the above equation, AR_*it*_ is the abnormal rate of return for stock *i* on day t of the incident period; R_*it*_ is the actual rate of return for stock *i* on day t of the incident period; and E(R_*it*_) is the expected rate of return for stock *i* on day t of the incident period.


$$\begin{array}{l} AAR_{it}=\bar{AR_{it}}=\frac{1}{N}\overset{N}{\underset{i=1}{\sum }}AR_{it} \end{array}$$

In order to more intuitively show the impact of the incidents on stock value, while studying the abnormal rate of return of stocks during the incident period, it is also necessary to calculate the cumulative abnormal rate of return of sample firms CAR_i_(t_1_,t_2_),T_1_ < t_1_ ≤ t_2_ ≤ T_2_, according to accumulated period of time (shown as follows).


$$\begin{array}{l} CAR_{i}\left(t_{1,\;}t_{2}\right)=\overset{t_{2}}{\underset{t=t_{1}}{\sum }}\bar{AR_{it}} \end{array}$$

According to the known average abnormal rate of return, the time can be further aggregated to obtain the cumulative average abnormal rate of return of individual stocks in the incident period (shown as follows).


$$\begin{array}{l} CAAR=\bar{CAR_{i}\left(t_{1,\;}t_{2}\right)}=\frac{1}{N}\overset{t_{2}}{\underset{t=t_{1}}{\sum }}\bar{AR_{it}} \end{array}$$

In the above equation, CAAR represents the cumulative average abnormal rate of return, and N represents the number of days.

Through the above calculation, the cumulative abnormal rate of return and average cumulative abnormal return of the sample in the incident period can be obtained (see Table 2).

**Table 2:** AAR and CAAR of two types of sample firms during the incident period.

	**Crisis firm**		**Competitive firm**	
**Day of the incident**	**Average abnormal rate of return (AAR)**	**Cumulative average abnormal rate of return (CAAR)**	**Average abnormal rate of return (AAR)**	**Cumulative average abnormal rate of return (CAAR)**
T-10	-0.00715	-0.00715	-0.00079	-0.00079
T-9	-0.00145	-0.0086	0.000559	-0.00023
T-8	0.01028	0.001681	-0.0039	-0.00412
T-7	0.012063	0.013744	0.004702	0.000579
T-6	0.013732	0.027476	0.007657	0.008236
T-5	-0.008	0.019477	-0.00426	0.003978
T-4	-0.00484	0.014634	0.000593	0.004571
T-3	0.011081	0.025715	0.001348	0.005919
T-2	-0.04837	-0.02265	0.001277	0.007196
T-1	-0.08899	-0.11165	-0.02452	-0.01733
T0	-0.15027	-0.26191	-0.03778	-0.05511
T+1	-0.12493	-0.38684	-0.05655	-0.11166
T+2	-0.0558	-0.44192	-0.03268	-0.14434
T+3	-0.0195	-0.46177	-0.01214	-0.15648
T+4	-0.0095	-0.47142	0.007338	-0.14914
T+5	-0.0158	-0.48722	-0.00328	-0.15242
T+6	-0.01418	-0.50139	0.004599	-0.14782
T+7	-0.0233	-0.52492	-0.00015	-0.14797
T+8	0.01805	-0.50692	-0.00638	-0.15435
T+9	0.005681	-0.50124	-0.00301	-0.15735
T+10	-0.00976	-0.511	0.00039	-0.15693

**Figure 1:**
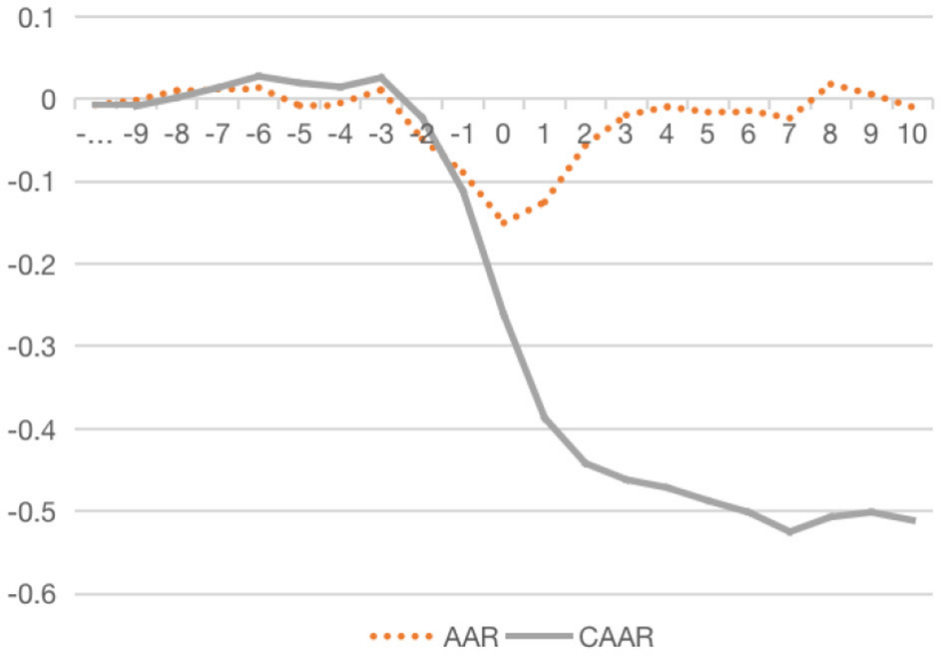
AAR and CAAR of crisis firms during the incident period.

Figure 1 shows the average abnormal rate of return and cumulative average abnormal rate of return of crisis firms during the incident period. It can be obviously seen that the average abnormal rate of return fluctuates around the value zero, and the fluctuation range of negative values is greater than that of the positive values. Therefore, the occurrence of food safety incidents has a negative impact on the stock price earnings of crisis firms. The change in the rate of return on the day of the incident fell into the lowest trough, indicating that the market responded quickly after receiving the crisis information. However, from the overall sample of crisis firms, with efforts made in public relations, clarification and announcement by the crisis firms, the negative impact gradually decreases, and the cumulative average abnormal rate of return begins to rise gradually after T7.

**Figure 2:**
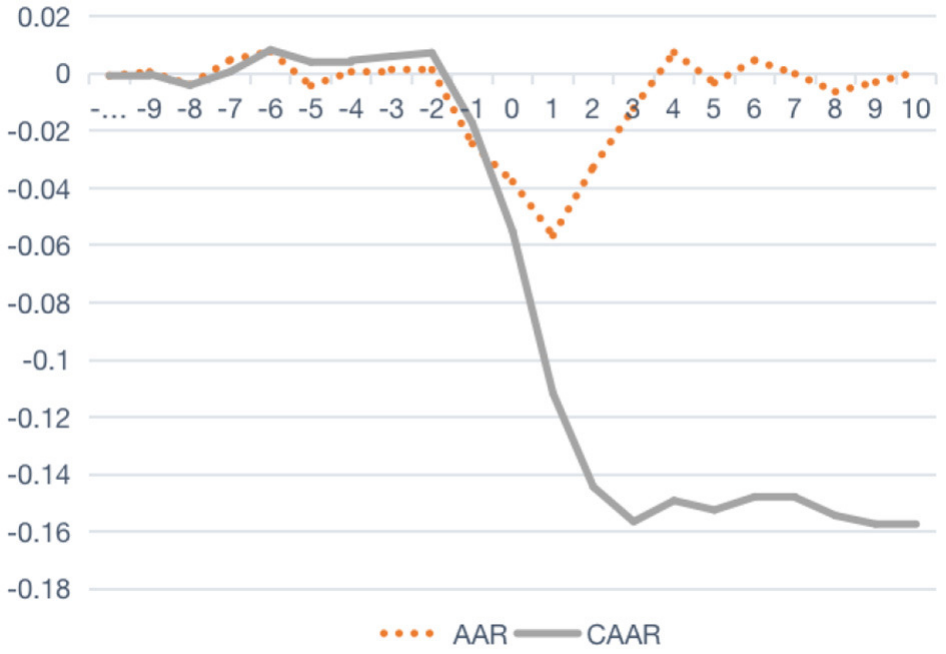
AAR and CAAR of the competitive firm during the incident period.

Figure 2 shows the average abnormal rate of return and cumulative average abnormal rate of return of competitive firms during the incident period. Due to the fact that competitive firms share the same or similar range of business with crisis firms, these competitive firms also, unpreventably, underwent suspicions by the market after the crisis incident; however, it can be seen that the lowest average abnormal rate of return of the competitive firm appears on day T1, which is 1 day after the appearance of the lowest value experienced by the crisis firm. In the meanwhile, the relative recovery period for the competitive firms is also shorter, and the average cumulative abnormal rate of return goes back to positive value on day T4.

#### T-Test

After obtaining the average abnormal rate of return and the cumulative average abnormal rate of return, a statistical test should be carried out to verify whether the incident has an impact on the stock value at a certain significance level. This paper uses *t*-test to determine whether the average cumulative abnormal rate of return is significantly different from 0. The results are shown in Table 3.

**Table 3:** Summary of significance of statistics T_AR_ of two types of sample firms during the incident period.

**Day of the incident**	**SAR of crisis firm**			**SAR of competitive firm**		
	**Mean (%)**	**T value**	***P*-value**	**Mean (%)**	**T Value**	***P*-value**
T-10	−0.7147	−1.757	0.122	−0.079	−0.264	0.793
T-9	−0.1452	−0.321	0.824	0.056	0.209	0.835
T-8	1.028	0.828	0.435	0.340	−1.010	0.317
T-7	1.206	1.392	0.207	3.065	1.208	0.232
T-6	1.373	0.790	0.456	0.766	1.906	0.061
T-5	−0.799	−1.048	0.330	−0.425	−1.237	0.221
T-4	−0.484	−1.219	0.262	0.059	0.207	0.837
T-3	1.108	1.842	0.009^**^	0.200	0.532	0.597
T-2	−4.837	−6.017	0.001^***^	0.128	0.536	0.594
T-1	−8.899	−12.581	0.000^***^	−2.452	−8.336	0.000^***^
T0	−15.03	−10.051	0.000^***^	−3.778	−6.996	0.000^***^
T1	−12.49	−6.843	0.001^***^	−5.655	−8.932	0.000^***^
T2	−5.508	−5.138	0.004^**^	−3.268	−10.775	0.000^***^
T3	−1.985	−4.342	0.010^*^	−1.214	−3.562	0.001^***^
T4	−0.964	−0.512	0.627	0.734	2.007	0.049^**^
T5	−1.579	−1.115	0.308	−0.328	−1.125	0.265
T6	−3.352	−3.605	0.100	0.460	1.588	0.118
T7	1.801	0.799	0.455	−0.015	−0.50	0.960
T8	0.986	0.713	0.548	−0.637	−2.292	0.025^**^
T9	0.568	0.688	0.517	−0.301	−0.928	0.357
T10	0.028	0.039	0.970	−1.744	−3.386	0.001

*** *P < 0.001*,

** *P < 0.05*,

* *P < 0.10*.

In the above table, mean value represents the standardized average abnormal rate of return, T value represents the standardized average abnormal rate of return, and *P*-value represents the level of significance. Through the test of the above statistics, it is found that during the incident period, food safety incidents have a significant impact on the stock prices of both crisis firms and competitive enterprises, and there is a negative contagion effect presenting on the competitive firms. Within [T-3, T3] days of the incident period, the normalized average abnormal rate of return is significantly different from 0 at the 90% confidence level; within [T-2, T2] days of the incident period, the normalized average abnormal rate of return is significantly different from 0 at the 95% confidence level, and even more significantly different from 0 at the 99% confidence level within the day before and after the incident. Therefore, hypothesis H1a is verified, i.e., food safety crisis of the firm has a negative impact on the market value of the crisis firm. As can be seen from Table 3, for competitive firms, the average cumulative abnormal rate of return shows an upward trend over time, so hypothesis H1b is also supported.

It can be noticed through the performance of the competitive firms that, within [T-1, T4] days of the crisis period, the average abnormal rate of return of the competitive firms after standardization is significantly different from 0 at the 95% confidence level, and is significantly negative. For these competitive firms, the average cumulative abnormal rate of return also shows an obvious negative relationship. Therefore, H1C is supported, i.e., the occurrence of food safety incidents of crisis firms has a spillover effect on their competitive firms, and seen from the overall picture, the contagion effect is greater than the competition effect.

The above quantitative research mainly explores our first research question, that is, after the occurrence of food safety incident, whether the crisis firm will bring about a spillover effect on the competitive firms. The results show that the crisis firm causes an obvious contagion effect on the competitive firms; however, with the development of the incident and the measures taken by the competitive firms, this contagion effect weakens in a gradual manner. Unfortunately, the utilization of the event study method can only explore issues such as the market value of competitive firms caused by the food safety incident; while factors affecting the change of market value of competitive firms involve various comprehensive facets. Therefore, based on the above research, our next step is to further study the impact of the characteristics and actions of competitive firms on their market value, i.e., whether the charitable donation of competitive firms before the crisis can regulate the crisis spillover effect; and if yes, how this spillover effect can be regulated, and whether the greater the donation amount is, the stronger this effect will be; in addition, in what direction the political connection of competitive firms will regulate the crisis spillover effect? Based on these two research questions, an empirical study will be carried out as below.

### Empirical Analysis

#### Model Design

In order to study the impact of charitable donations and political connections of competitive firms before the crisis on their average cumulative abnormal rate of return, a regression equation model is built for empirical analysis. The detailed model is shown as follows.


$$\begin{array}{l} PCAR_{i}=\alpha +\beta_{1}\;*\;Pol+\beta_{2}\;*\;ifDon+\beta_{3}\;*\;Pol\;*\;ifDon\\ \;+\beta_{4}\;*\;size+\beta_{5}\;*\;debt+\beta_{6}\;*\;CF\;+\beta_{7}\;*\;OC\\ \;+\beta_{8}\;*\;event+\xi \end{array}$$

In this equation, PCAR_*i*_ represents the average cumulative abnormal rate of return of competitive firms, where *i* = 1,10; Pol indicates the political connections of key executives of the competitive firm during the crisis period; if Don represents whether the competitive firm had made charitable donations in the previous year before the crisis occurred; size indicates the size of the competitive firm; Debt represents the asset liability ratio of the competitive firm; CF represents the cash flow of the competitive firm; OC represents the equity concentration of the competitive firm; incident is the incident identifier. β_i_ represents the regression coefficient to be estimated, where *i* = 1,2,3,…,8; ε is the random residual term.

#### Descriptive Analysis

This paper uses EViews 7.0 to carry out ADF test. It is found that the individual stock returns and relevant market returns of the sample firms remain series stationarity, therefore, corresponding correlation regression analysis can be carried out. Before the econometric analysis, descriptive statistics is conducted to observe the sample data.

Table 4 is based on the descriptive statistics of the variable data of 62 competitive firms. It is found that the average cumulative abnormal rate of return of competitive firms in the 1-day window period is significantly higher than that in the 10-day window period, indicating that with the continuous development of the incident, the negative crisis effect of the incident on competitive firms continues to weaken. From the data as regard to whether firms had made donations or not, the average value exceeds 0.5, indicating that most firms are willing to make charitable donations. However, from the perspective of donation intensity, different firms have shown relatively great disparity according to the data on previous donations.

**Table 4:** Descriptive statistics of variables of sample firms.

**Variable**	**Mean**	**Std. Dev**.	**Min**	**Max**
PCAR1	−0.007	0.011	−0.037	0.024
PCAR10	−0.001	0.009	−0.018	0.021
Don	7.202	6.347	0.000	17.925
IFDON	0.597	0.495	0.000	1.000
Pol	0.581	0.497	0.000	1.000
Size	21.826	1.105	19.411	24.332
Debt	0.429	0.187	0.058	1.063
cf	0.290	0.288	0.000	1.455
oc	58.273	17.091	17.820	88.410
CASE	4.952	2.161	1.000	8.000

#### Correlation Analysis

Before regression analysis of the equation, the correlation of each variable must be determined through correlation analysis. Correlation analysis aims to test whether there is a relationship between each variable, and uses correlation coefficient to quantify the strength of this relationship.

According to Table 5 showing the correlation coefficient between sample variables, it is found that the core explanatory variables (“donate or not” and “donation intensity”) are significantly and positively correlated with the explained variable (the average cumulative abnormal rate of return of competitive firms) in the two incident windows at the confidence level of 99%; also, political connection is positively correlated with the explanatory variable in the 1-day window period, and negatively correlated with the explanatory variable in the 10-day window period, but not significant enough. After adding other control variables, we further study the relationship between political connection and average cumulative abnormal rate of return of competitive firms. The average cumulative abnormal rate of return of competitive firms in the 1-day window period is significantly and positively correlated with that in the 10-day window period, and there is also a significant positive correlation between “donate or not” and “donation intensity.”

**Table 5:** Correlation coefficients of variables of sample firms.

**Variable**	**PCAR1**	**PCAR10**	**Don**	**IFDON**	**Pol**	**Size**	**Debt**	**cf**	**oc**	**CASE**
PCAR1	1.000									
PCAR10	0.392^***^	1.000								
Don	0.480^***^	0.295^**^	1.000							
IFDON	0.474^***^	0.301^**^	0.940^***^	1.000						
Pol	0.128	−0.114	0.253^**^	0.234^*^	1.000					
Size	−0.020	−0.142	0.186	0.096	0.310^**^	1.000				
Debt	0.029	0.009	0.083	0.066	0.284^**^	0.188	1.000			
cf	−0.205	−0.134	−0.083	−0.091	−0.013	−0.023	−0.517^***^	1.000		
oc	0.078	−0.016	0.085	0.115	0.003	0.371^***^	−0.108	0.038	1.000	
CASE	−0.042	−0.362^***^	−0.207	−0.141	0.072	−0.094	0.171	−0.030	0.075	1.000

*** *P < 0.001*,

** *P < 0.01*,

* *P < 0.05, two-tailed test*.

#### Regression Analysis

It can be seen from Table 5 that the correlation coefficients of all core explanatory variables, explained variables and control variables to be regressed are < 0.700 except for the two variables “donate or not” and “donation intensity”; however, these two variables (“donate or not” and “donation intensity”) will not enter the regression equation together, therefore, the problem of multicollinearity between each variable is not serious. In order to further diagnose the multicollinearity problem of the model, tests of tolerance and variance expansion factor are conducted. The results show that the maximum value of VIF is no more than 4.8, and the value of TOL is far >0.1, so it can be determined that the model has no multicollinearity problem, and regression analysis can be carried out. In order to explore the impact of political connections and charitable donations on the average cumulative abnormal rate of return of competitive firms in different incident window periods, a 1-day window period was selected to test the timeliness of market response and a 10-day window period is selected to strengthen the robustness.

Table 6 summarizes the regression results of the model in the 1-day and 10-day window periods. Different incident identifiers are all controlled in the model to avoid the deviation of results caused by sample data not being in the same incident. Model 1 shows the regression result of the core explanatory variable (“donate or not”) and the explained variable (average cumulative abnormal rate of return of competitive firms) after controlling each control variable. It is found that for competitive firms, the coefficients of “donate or not” before the crisis and their average cumulative abnormal rate of return are positively significant at the levels of 95% and 99%, respectively. This means that whether the competitive firms have made charitable donations will effectively weaken the crisis spillover effect during the event period. Thus, Hypothesis H3a holds. In addition, all control variables have passed the significance test, which shows that these control variables have played an effective role in the equation. Model 2 is the model showing relationship between political connection as the core explanatory variable and the average cumulative abnormal rate of return of competitive firms as the explained variable. The results show that there is no significant relationship between the political connection of competitive firms and their average cumulative abnormal rate of return, which is consistent with what is proposed in hypothesis H2a. Model 3 shows the result when the core explanatory variable (political connection) and “donate or not” are entered into the model at the same time, the result of which is generally consistent with the previous conclusions.

**Table 6:** The results of regression analysis of [−1,1] and [−10,10] window period.

	**[−1,1] window period**			**[−10,10] window period**		
**PCAR1**	**Model 1**	**Model 2**	**Model 3**	**Model 1**	**Model 2**	**Model 3**
IFDON	0.00324^**^		0.00310^**^	0.00565^***^		0.00421^***^
	(0.00021)		(0.00025)	(0.00013)		(0.00023)
Pol		0.00132	0.00099		0.00180	0.00096
		(0.00141)	(0.00175)		(0.00092)	
Size	0.00019^**^	0.00023^**^	0.00031^***^	0.00029^**^	0.00027^**^	0.00031^***^
	(0.00009)	(0.00009)	(0.00009)	(0.00018)	(0.00098)	(0.00009)
Debt	−0.00344	−0.00524	−0.00504	−0.00101^**^	0.0117^**^	0.0118^**^
	(0.00823)	(0.0109)	(0.00866)	(0.00433)	(0.00486)	(0.00494)
cf	0.00173^*^	0.00212^*^	0.00184^*^	0.00200^**^	0.00185^*^	0.00197^*^
	(0.00117)	(0.00152)	(0.00164)	(0.00117)	(0.00135)	(0.00134)
oc	0.0008^**^	0.0010^*^	0.0012^**^	0.0012^**^	0.0009^***^	0.0011^**^
	(0.00003)	(0.00007)	(0.00047)	(0.00043)	(0.00001)	(0.00052)
Constant	0.0256	0.0305	0.0314	0.00271	−0.00386	−0.00360
	(0.0255)	(0.0289)	(0.0246)	(0.0185)	(0.0211)	(0.0212)
Case effect	Yes	Yes	Yes	Yes	Yes	Yes
*R*-squared	0.574	0.491	0.581	0.633	0.610	0.646

*** *P < 0.001*,

** *P < 0.01*,

* *P < 0.05, two-tailed test, two tailed test; the values listed in the chart are regression coefficients; the values in parentheses are standard errors*.

#### The Heckman Two-Stage Model

The above regression analysis has verified that charitable donation actions of competitive firms will alleviate the negative effects of crisis spillover. However, the difference made by the positive roles of different levels of donation intensity remains to be explored. In view of this, this study applies the Heckman two-stage model for regression analysis. The application of the Heckman two-stage model mainly involves consideration of the following two aspects. First, this model takes the virtual variable of “donate or not” as the explained variable. When there is donation made by the competitive firm before the crisis, the variable is set to be 1, otherwise it is set to be 0. According to the above, a probit model recorded as model 1 is established to study the impact of donation made by the competitive firm before the crisis on the crisis spillover effect. Second, model 2 or 3 are established on the premise that the competitive firm has made charitable donation, that is, the virtual variable of ifDon is 1; then the variable of real charitable donation intensity “Don” is used as the explanatory variable to examine the specific impact of the intensity of the charity donation made by the competitive firm on the spillover effect.

The regression results are shown in Table 7. Model 1 shows that the political connection of the competitive firm is positively correlated with whether it donates at the 95% significance level, which means that political connection of the competitive firm will enhance the probability of its charitable donation. Firms may carry out public welfare donation with its own strategic concerns in order to establish effective ties with the government. Model 2 shows that in the 1-day window period, the intensity of charitable donations is positively correlated with the average cumulative abnormal rate of return of competitive firms at a significant level of 95%, which means in the 1-day window period, the more charitable donations made by the competitive firms, the more effective it is to alleviate the negative crisis spillover effect. Model 3 shows similar result to that of model 2 in that the intensity of charitable donation is positively correlated with the average cumulative abnormal rate of return of competitive firms at a significant level of 99% in the 10 day window period, which means compared with the 1-day window period, the effect of the intensity of donation of competitive firms on alleviating the negative spillover effect of the crisis is more obvious in the 10 day window period. Therefore, H3b and H3c both hold.

**Table 7:** Summary of the regression results of Heckman two-stage model.

	**Model 1**	**Model 2**	**Model 3**
**VARIABLES**	**IFDON**	**PCAR1**	**PCAR10**
Don		0.00375^**^	0.00591^***^
		(0.00124)	(0.00001)
Pol	0.681^**^	0.00164	0.00175^*^
	(0.072)	(0.00113)	(0.00107)
Size	0.0327^**^	0.00036^***^	0.00026^**^
	(0.0173)	(0.00007)	(0.00014)
Debt	−0.372	0.0198	0.0284
	(1.110)	(0.379)	(0.179)
cf	0.554^**^	0.00173^*^	0.00243^**^
	(0.182)	(0.00154)	(0.00087)
oc	0.00996^***^	0.0011^**^	0.0013^***^
	(0.0009)	(0.00016)	(0.00003)
Constant	0.318	0.116	0.0731
	(3.487)	(1.186)	(0.559)
Case effect	No	Yes	Yes

*** *P < 0.001*,

** *P < 0.05*,

* *P < 0.10*.

In terms of control variables, it can be seen in model 1 that the size, cash flow and equity concentration of the firm are significantly positively correlated with whether the firm donates, indicating that the larger the firm size, the more sufficient the cash flow and the higher the equity concentration, the more likely the firm is to make charitable donations. In models 2 and 3, firm size, cash flow and equity concentration are positively correlated with the average cumulative abnormal rate of return of competitive firms, which is consistent with the results of previous research.

## Conclusions and Implications

### Research Conclusions

Through methods such as event study, regression analysis and Heckman two-stage model, this paper explores the crisis spillover effect of food safety incidents on firms in the food industry, and conducts an in-depth study on the impact of political connections and charitable donations of competitive firms on their average cumulative abnormal rate of return. It is found that the occurrence of food safety incidents will have a negative impact on the market value of crisis firm, but with the passage of the incident, the effect of the negative impact continues to weaken. The main reason is that after the incident, crisis firms will take effective countermeasures, such as clarification, apology, distinction, etc.; in addition to the fact that consumers' attention is limited after all, both consumers and the media will eventually pay less attention to the incident; and with involvement of other influencing factors, the negative effects will be weakened with the passage of time. Furthermore, the occurrence of food safety incidents will also exert an impact on competitive firms inside and outside the industry. The results show that the overall crisis spillover effect of food safety incidents on competitive firms is a negative one, to be exact, a contagion effect. It is noticed from the overall sample that, in the first 3 days of the incident, due to the possible early disclosure of the incident information and the small-scale spread of information related to the incident, consumers will have a sense of distrust of competitive firms in the same industry. However, with the gradual unfolding of the incident, consumers and investors will come to understand that the incident may only be caused by only an individual firm or a small group of firms due to some illegal operation. After such clarification of facts, the negative impact of the incident will be gradually weakened.

In addition, our research results show that the political connection of competitive firms before the crisis has no significant effect on their average cumulative abnormal rate of return during the crisis period. In other words, political connections of competitive firms play no significant positive or negative role in influencing average cumulative abnormal rate of return during the crisis period. This may due to the fact that there are too few samples collected in this paper, and the implicit impact of political connection on the yield of the competitive firms is yet to be accurately captured. However, the donation actions made by the competitive firms before the crisis will increase their cumulative abnormal rate of return during the crisis, meaning that the charitable donation actions of competitive firms will cause significant positive effect. The greater the intensity of such charitable donation made by the competitive firms before the crisis, the more positive the impact on their average cumulative abnormal rate of return; and the significance of this impact increases with the extension of the incident window.

### Managerial Implications

On the basis of quantitative and empirical research, this paper verifies the existence of crisis spillover effect, discusses the relationship between political connection, charitable donation and crisis spillover effect, and reaches a few practical conclusions. Although only the food industry is explored, this study still provides useful implications for firms in various industries in avoiding negative spillover effect of crisis and striving for positive spillover effect in the future. To be specific, this study provides managerial implications at both industrial and firm levels.

At the industrial level, the industry should strengthen the norms of industrial autonomy. Food industry, especially, is an industry with a high incidence of crisis. Once a food safety incident occurs, it will not only put the crisis firm into business meltdown, but also affect a large number of competitive firms in the same industry and bring a significant impact to the whole industry. In detail, trade associations should play an active role in strengthening the autonomy and standardization of the industry, constructing the review mechanism of the industry, and guiding the whole industry to form a high-quality business model, which takes quality as the foundation and services as the guarantee.

Seen from the firm level, first of all, firms should lay absolute emphasis on product quality. Quality is the foundation of a firm's survival, and ensuring product quality should be considered the most fundamental and prioritized obligation for a firm. To be specific, firms should establish an effective safety management system to ensure product quality from various aspects such as the sources of raw materials, production, processing, distribution and so on. Second, attention should be paid to information related to industrial crisis. Firms should not develop themselves behind closed doors; instead, attention should always be paid to the dynamics of competitive firms through sensing and collecting all kinds of information related to potential and existing crisis, grasping the public opinion guidance of the media and the public, and formulating and taking effective responses in a timely manner. Third, firms should actively undertake corporate social responsibilities. Our research conclusion shows that actions related to corporate social responsibilities in such form as charitable donation can effectively reduce the negative impact during the crisis. Through actively shouldering social responsibilities, firms are able to establish a decent corporate image, play a positive external role in the crisis, thus, protecting or buffering the damage of the crisis.

### Limitations and Future Prospects

The limitations of this study are mainly as follows. From the perspective of sample size, only a limited number of incidents of listed companies are used as our samples, which has brought difficulty especially in the observation stage. Also, since most of the incidents studied are individual and independent ones, so the specific performance of systematic incidents in different incident windows can seldom be displayed. Therefore, according to the above-mentioned issues, subsequent research can expand its scope to include not only the domestic A-share market, so as to obtain more samples. In addition, we need to control the types of events in different classifications in order to further study the corresponding disparities.

Furthermore, from the perspective of research scope, the sample of competitive firms selected in this paper are firms with the same or similar main businesses in the industry. However, the scope of spillover effect is actually far more than this. Upstream and downstream firms in the same industry and related firms outside the industry will also be implicitly implicated. Future studies can be conducted through expanding the research scope and classifying the type of incidents in order to explore more distinctive scenarios. In addition, the impact of upstream and downstream firms within the industry and relevant firms outside the industry can also be studied. From the perspective of research methods, this paper mainly uses the event study method to test the spillover effect, and then verifies the existence of such spillover effect through constructed statistics, namely, standardized abnormal rate of return. However, due to the immaturity of China's stock market, our research results may also be affected by other factors within the relatively clean event window. For example, there may be a deviation in calculating the cumulative abnormal rate of return of individual stocks. For the sake of robustness, this issue should be further considered in the future. Therefore, other statistical methods are suggested to be utilized in the future.

Last but not the least, from the perspective of research content, this paper also studies the impact of such variables as political connection and charitable donation on crisis spillover effect; other factors that may also cause fluctuations in stock prices should be taken into account such as the impact scope and significance of the food safety incidents, the respective industrial status of crisis firms and competitive firms and so on. However, due to the difficulty of obtaining these control variables, these variables are not added in this paper for the sake of time and energy. In the future, more firms should be considered and appropriate indicators should be selected for classification-based researches.

## Data Availability Statement

The raw data supporting the conclusions of this article will be made available by the authors, without undue reservation.

## Author Contributions

RX was in charge of the formulation of the general research topic, the construction of the research framework, and the proposing of the theoretical hypothesis. MW generally contributes to the construction of the theoretical framework based on the in-depth accumulation of a large volume of literature reading and analysis and contributes a lot in the data analysis process. LL was in responsible for the overall development of this study, including the selection of research angle, research dimensions, the planning of sample collection, data analysis and proof-reading, and polishing of the whole paper. DW was in charge of data collection, analysis of this study, and responsible for all the procedures taken during data collection. All authors contributed to the article and approved the submitted version.

## Funding

This research is financially supported by the National Natural Science Foundation of China (Grant number: 71773115), the National Natural Science Foundation of China (Grant number: 71973129), and the Major Bidding Project of National Social Science Fund of China (Grant number: 20&ZD124).

## Conflict of Interest

The authors declare that the research was conducted in the absence of any commercial or financial relationships that could be construed as a potential conflict of interest.

## Publisher's Note

All claims expressed in this article are solely those of the authors and do not necessarily represent those of their affiliated organizations, or those of the publisher, the editors and the reviewers. Any product that may be evaluated in this article, or claim that may be made by its manufacturer, is not guaranteed or endorsed by the publisher.
